# New species, new records and new morphological characters of the genus *Tillicera* Spinola from China (Coleoptera, Cleridae, Clerinae)

**DOI:** 10.3897/zookeys.122.1457

**Published:** 2011-08-11

**Authors:** Ganyan Yang, Olivier Montreuil, Xingke Yang

**Affiliations:** 1 Key Laboratory of Zoological Systematics and Evolution, Institute of Zoology, Chinese Academy of Sciences, Beijing, 100101, P.R. China; 2 Graduate School, Chinese Academy of Sciences, Beijing, 100039, P. R. China; 3 UMR 7205, Département de Systématique et Évolution, Muséum National d’Histoire Naturelle, CP 50, 57 rue Cuvier, F-75231 Paris,Cedex 05, France

**Keywords:** Coleoptera, Cleridae, *Tillicera*, China, new species, sensillum

## Abstract

Two new species of the genus *Tillicera* Spinola, 1841 from China are described and illustrated: *Tillicera sensibilis* **sp. n.** from Yunnan (also from Myanmar, Thailand and Laos) and *Tillicera wenii* **sp. n.** from Taiwan. *Tillicera bibalteata* Gorham, 1892, *Tillicera hirsuta* (Pic, 1926) and *Tillicera michaeli* Gerstmeier & Bernhard, 2010 are newly recorded from China. *Tillicera auratofasciata* (Pic, 1927) is newly recorded in some provinces of China. A key to species of the genus from China is provided. Relationships between species are discussed with emphasis on characters of male phallus, female internal reproductive organs and pit-like sensilla in male terminal antennomere, which is discovered in *Tillicera* for the first time. The present generic definition of *Tillicera* is discussed as well. Photos of terminalia of the previously known species are also provided for comparison.

## Introduction

Fifteen species of the oriental genus *Tillicera* Spinola, 1841 were recognized by Gerstmeier and Bernhard (2010) whose thorough generic revision, with the exception of *Tillicera assamensis* Stebbing, 1907 from Assam, India, was based on first hand examination of primary types. Two species occur in China: *Tillicera auratofasciata* (Pic, 1927) from Xizang and *Tillicera cleroides* Gorham, 1892 from Yunnan. In the course of our studies of material from several major Chinese collections and additionally some European museums, two new species were discovered: *Tillicera sensibilis* sp. n. from Yunnan (also from Myanmar, Thailand and Laos) and *Tillicera wenii* sp. n. from Taiwan; both of which exhibiting a similar habitus to that of *Tillicera auratofasciata* (Pic, 1927), *Tillicera javana* Spinola, 1844 and *Tillicera soror* Schenkling, 1902, but with significantly different aedeagi. Furthermore, new distribution records of some previously known species were found.

Gerstmeier and Bernhard (2010) did not separate the tegmen and phallus, and thus some systematically significant characters of the phallus were not quite clear. In the present study the tegmen and phallus are separated apart and fine structures of the phallus are carefully compared.

For the first time, high resolution color photographs of female internal reproductive organs of Cleridae are provided. The female internal reproductive organs of all the species from China are compared except for *Tillicera wenii* due to a lack of specimens. And for the first time, pit-like sensilla on the male terminal antennomere in *Tillicera* is discovered (in dorsal view). Comparative photographs of this structure are presented for *Tillicera auratofasciata* and the two new species described herein. The purpose of this paper is to describe two new species, present new distribution records for previously known species, discuss the relationships among species and the generic definition of the genus, and provide some new morphological characters which might better facilitate further systematic study of the genera related to *Tillicera* and *Clerus*.

## Material and methods

Materials examined are deposited in the following collections; abbreviations as shown in the text:

CAU	China Agricultural University, Beijing, China

INCA	Insects collection of INCA Science Ltd., Chongqing, China

IZAS	Institute of Zoology, Chinese Academy of Sciences, Beijing, China

MCSN	Museo Civico di Storia Naturale, Genova, Italy

MHBU	Museum of Hebei University, Baoding, Hebei, China

MNHN	Muséum National d’Histoire Naturelle, Paris, France

NMNS	National Museum of Natural Science, Taiwan, China

NMPC	National Museum, Prague, Czech Republic

RGCM	Roland Gerstmeier Collection, Munich, Germany

SHNU	Department of Biology, Shanghai Normal University, China

SWFU	Southwest Forestry University, Kunming, China

Whole male abdomens were removed from the body with fine forceps, treated with 10% KOH solution at room temperature for 8–12 hours. Terminalia were prized apart, rinsed and examined in 70% ethanol, then photographed in glycerol and eventually stored within glycerol in genital vials which were pinned below specimens. Female internal organs were all dissected from dry specimens. The female specimens of *Tillicera auratofasciata* and *Tillicera sensibilis*, collected in 2009 and 2008 respectively, were killed by ethyl acetate then preserved in ethanol and pinned later. Whole female abdomens were removed from the body with fine forceps, treated with 10% KOH solution at room temperature for 12–16 hours. Female reproductive organs were prized apart, rinsed in 70% ethanol, stained with chlorazol black E saturated solution in 70% ethanol for 40 seconds, photographed in 70% ethanol then mounted on a plastic slide in euparal, which was pinned below specimens. Antennae were removed from the head (segments 3–11 of *Tillicera auratofasciata* and *Tillicera sensibilis*; 10–11 of *Tillicera wenii*), treated with 10% KOH solution at room temperature for 48 hours and rinsed with water before being photographed.

Habitus images were captured using a Canon 450D digital camera with Canon Macro 100 mm lens; genitalia were captured by a Canon 450D digital camera fitted to a Nikon SMZ–1500 stereoscopic dissecting microscope; antennae were captured by a Nikon digital Sight DS–SM camera fitted to a Nikon SMZ–1500 stereoscopic dissecting microscope controlled by ACT–2U software. Series of partially focused photographs were taken and then combined using Helicon Focus software, and finally processed with Adobe Photoshop software. Line drawings of pronota were made from color photographs with the software Adobe Illustrator.

Measurements were made under a stereo microscope using an ocular micrometer. Body length is the linear distance from labrum to elytral apex. Body width is the maximum width across elytra. Lengths and widths of terminal antennomeres were measured as in Fig. 29.

Terminology mostly accorded with Ekis (1977). When describing the phallus, we do not consider the natural orientation, but define the dorsal face as the position where the median orifice (*sensu* Sharp and Muir 1912) opens, (and the opposite as the ventral face), and thus, each phallic plate has a dorsal margin and a ventral margin, and the membrane connecting the two ventral margin called phallic ventral membrane. The pulvillus of tarsomeres is abbreviated to P; for example ‘P1’ indicates pulvillus of the tarsomere 1, and ‘pro-P1’ indicates the pulvillus of the protarsomere 1 (clerids usually have pulvilli present on tarsomeres 1–4, while tarsomere 5 is slender and without a pulvillus).

## Taxonomy

### Key to species of Tillicera Spinola from China

**Table:** 

1	Pronotum gradually narrowed after subapical depression, the widest part of the pronotum proper (*sensu*Ekis 1977) is in the extreme front (Fig. 3); anterior margin of pronotum with a band of dense, golden, decumbent setae	*Tillicera cleroides* Gorham
–	Pronotum swollen after subapical depression, the widest part of the pronotum proper (*sensu*Ekis 1977) is near the middle (Fig. 4); anterior margin of pronotum without a band of dense, golden, decumbent setae	2
2	Elytra with two bands of golden decumbent setae, one just before middle, another near the apex, sometimes few additional sparse golden setae present at base; pro- and mesotibiae never with longitudinal carinae, metatibiae sometimes with indistinct rudiment of carinae	3
–	The middle golden or light yellow band on elytra split, forming a middle backward opened lunate band and two laterally transverse spots (Figs 1–2); the subapical band as in the previous species; all tibiae with distinct longitudinal carinae	5
3	Elytral base with a distinct tuberosity, sometimes with some sparse golden setae around the tuberosity; integument from which the two bands of golden setae originate black	*Tillicera michaeli* Gerstmeier & Bernhard
–	Elytral base without a distinct tuberosity, never with sparse golden setae either; integument from which the two bands of golden setae originates yellow	4
4	Apex of elytra emarginate; elytral base with longitudinal rows of tubercles present on intervals	*Tillicera hirsuta* (Pic)
–	Apex of elytra not emarginate; elytral base without such tubercles	*Tillicera bibalteata* Gorham
5	The pattern-forming setae on elytra light yellow; meta-P1 evident but unlobed, meta-P2 distinctly bilobed in apex (Fig. 40); pit-like sensilla of male terminal antennomere smaller and deeper, indistinct in magnification 100× before treated with KOH solution (Fig. 31)	*Tillicera wenii* sp. n.
–	The pattern-forming setae on elytra golden; Meta-P1 absent, meta-P2 just vestigeal and not clear (Fig. 39); pit-like sensilla of male terminal antennomere larger and shallower, clearly distinct in magnification 100× before treated with KOH solution (Figs 29–30, 32–33)	6
6	Pubescence on metaepisternum and metasternum yellow, almost as thick as golden decumbent setae on elytra; metaepisternum black, at least the lateral part of metasternum black; length to width ratio of terminal antennomere of both sexes < 1.5 (Fig. 29)	*Tillicera auratofasciata* (Pic)
–	Pubescence on metaepisternum and metasternum white, distinctly far thinner than light yellow decumbent setae on elytra; metasternum and metaepisternum always earth-yellow; length to width ratio of terminal antennomere of both sexes > 1.5 (Fig. 30)	*Tillicera sensibilis* sp. n.

#### Tillicera auratofasciata

(Pic, 1927)

http://species-id.net/wiki/Tillicera_auratofasciata

Figs 5–12 29, 32 34–37

Pseudoclerops auratofasciata  Pic, 1927: 8 (Chapa, N Vietnam).
Tillicera auratofasciata : Pic, 1934: 133.

##### Diagnosis.

Distinguishable from superficially similar specimens of *Tillicera sensibilis* by body stouter (length to width ratio of elytra: male about 2.04, female 2.11), length to width ratio of terminal antennomere of both sexes evidently below 1.5, metaepisternum black, at least the lateral part of metasternum black, pubescence on metaepisternum and metasternum yellow and almost as thick as golden decumbent setae on elytra, dorsal sinus of tegmen triangular, ventral sinus digitiform, and dorsal margin of phallus with large denticles extending from anterior half to posterior sclerotized area; and distinguishable from *Tillicera wenii* by pattern-forming setae on elytra more gold colored,  metaepisternum black, at least the lateral part of metasternum black, pubescence on metaepisternum and metasternum yellow and almost as thick as golden decumbent setae on elytra, meta-P1 absent and meta-P2 just vestigeal, pit-like sensilla of male terminal antennomere larger and shallower (distinct in magnification 100× before treated with KOH solution) (Figs 29, 32), paramere not pointed, dorsal sinus triangular, ventral sinus digitiform.

##### Supplemental description.

Elytra: Length to width ratio: male about 2.04, female about 2.11.

Terminal antennomere: Length to width ratio of both sexes below 1.5; male with a shallow pit-like sensillum present at basal third, diameter 0.08 mm (clearly distinct in magnification 100× before and after treated with KOH solution) (Figs 29, 32).

Male terminalia: Ratio of length of paramere to whole tegmen 0.17: 1, paramere thumb-like in dorsal view, dorsal sinus triangular, ventral sinus digitiform (Figs 5–6); phallic plate with large denticles along dorsal margin and fine granular structures on posterior sclerotized area (denticles extending from anterior half to posterior sclerotized area; number of denticles can vary between left and right phallic plate and between individuals but whole length of denticles is consistent) (Figs 7–8, 12); phallic ventral membrane with one line of file-like structures on each side (Fig. 7); spicular fork (Fig. 9); tergum VIII (Fig. 10), sternum VIII (Fig. 11).

Female reproductive organs: Vagina swollen, base of bursa copulatrix narrower than posterior end of vagina; swollen zone between vagina and spermathecal duct larger than that in *Tillicera sensibilis*; length of bursa copulatrix three times as long as spermatheca; spermathecal gland attached to basal fourth of spermatheca (Figs 36, 37); both dorsal and ventral lamina of ovipositor undivided (Figs 34, 35).

##### Distribution.

China: Yunnan (new record), Guangxi (new record), Hainan (new record), Xizhang; Thailand; Laos; Vietnam.

##### Material examined.

 **Lectotype** designated here: female, “Type [printed]/Type [handwritten by Pic]/Tonkin, Chapa, 27.VI.1918, Jeanvoine/*Pseudoclerops auratofasciatus* n. sp. [handwritten by Pic]/*Tillicera auratofasciatus* Pic/Museum Paris, Coll. M. Pic/HOLOTYPE *Tillicera auratofasciata* (Pic, 1927), Revision 2009, Gerstmeier & Bernhard” (MNHN).

##### Remarks. 

The name-bearing type was not fixed in the original publication and there was no imply how many type series was. For the purpose to fix the name-bearing type, the lectotype is designated here, which is the same specimen noted as "holotype" in Gerstmeier & Bernhard (2010).

##### Other material examined.

 **China**: 1 male, Yunnan, Xishuangbanna, Cheli, Shihuiyao, alt. 700m, 1957.IV.27, leg. WANG Shuyong (IZAS); 1 female, Yunnan, Xishuangbanna, Mengla, alt. 620–650m, 1959.V.20, leg. ZHANG Facai (IZAS); 1 female, Yunnan, Xishuangbanna, Mengla, Menglun, No. 55 area, 21.9650°N, 101.2099°E, ca 630m, 2009.VIII.2, leg. SHI Hongliang, by beating (IZAS); 1 female, Yunnan, Nabanhe N.R. Xiaonuoyouxiazhai, alt. 1700m, 2009.V.8, leg. HU Jiayao & YIN Ziwei (SHNU); 1 male, Guangxi, Luocheng, Yuxi station, alt. 400—700m, 2003.VII.29–31, leg. YANG Xiujuan (MHBU); 1 female, Hainan, Jianfengling Nature Reserve, 2007.V, leg. DING Liang (IZAS).

**Vietnam:** 1 female, Tonkin, Mt. Bavi, alt. 800–1000m, 1941.VII, leg. A. De Cooman (IZAS); 2 females, Tonkin, Hoa–Binh, leg. A. De Cooman (IZAS); 1 male, Tam Dao, 930m, Vinh Phu Prov., N. Vietnam, 1~8–V–1998, Y. Arita leg.(MNHN); 2 females, Tam Dao, Vinh Phu Pro., N. Vietnam, Apr. –7 May 1996, Native leg. (MNHN); 1 male, Cuc Phuong, Ninh Binh Prov., N. Vietnam, 5–VI–1997, Y. Okushima leg. (MNHN); 1 male, TamDao, N. Vietnam, VIII. 1999 (RGCM); 1 male, N-Vietnam, Tam Dao, V.90, Dembicky leg. (RGCM); 1 male, 1994.May.2—8, TamDao, (N. Vietnam), local-col. (RGCM); 1 female, Mt. Tam Dao, Vinh Phu, N. Vietnam, V. 1998 (RGCM).

**Thailand:** 1 male, N. Thailand, Meo Village, near Chiang Mai, V. 1988 (RGCM); 1 female, Mt. Doi Pui, 1400–1500m, Chiang Mai, N. Thailand, 22.V.1982, T. Shimomura leg. (MNHN); 1 female, Thailand, Chiangmai, Doi Pui, 26.V.1985 (RGCM); 1 female, N. Thailand, Doi Inthanon Nat. Res., 1250m, 22—31.V.2008, S. Murzin leg.

**Laos:** 1 male, Laos, Umg. Vientiane, III.—VI.1963 (RGCM);

#### Tillicera sensibilis

G.Y. Yang & X.K. Yang sp. n.

urn:lsid:zoobank.org:act:B341B5D4-FDEE-4E60-B2BE-01A560A4F6C1

http://species-id.net/wiki/Tillicera_sensibilis

**Figures 1–2.**
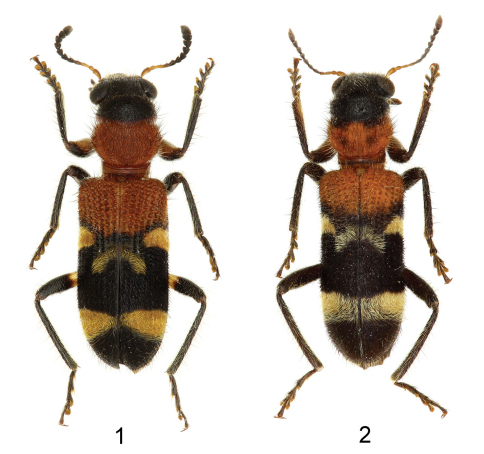
Habitus **1** *Tillicera sensibilis* sp. n., paratype, from Yingjiang, Yunnan **2** *Tillicera wenii* sp. n., holotype, from Lalashan, Taiwan.

Figs 1 4 13 20 30, 33 38 39 

##### Holotype

male. China, Yunnan, Yingjiang, Tongbiguan, Jianbian, Jingzhuzhai, 24.6119°N, 97.6153°E, ca 1420m, 2008.VI.4, leg. SHI Hongliang, by light trap. Specimen pin mounted; locality label (original in Chinese); label with information on dissecting number (56#), dissector and date; holotype label; plastic vial with abdomen and aedeagus (IZAS). **Paratypes**. **China**: 2 males, 4 females, same locality data as holotype (1 male preserved in RGCM, others in IZAS); 1 male, Yunnan, Yingjiang, Xima, Menglai River second class hydroelectric power station, 24.7840°N, 97.6749°E, ca 1470m, 2008.VI.6, leg. SHI Hongliang, by light trap (IZAS); 1 female, same data but 2009.V.27–29, leg. ZHANG Weiwei (INCA); 1 male 1 female, Yunnan, Ruili, Dengga to Mafengshan, 23.9529°N, 97.5981°E – 23.9449°N, 97.5565°E, alt. 927–1207m, 2009.VIII.10, leg. SHI Hongliang, by beating (IZAS); 1 female, Yunnan, Yongde, Daxueshan, Manlai, Huataoshu, 1520m, 2002.VI.17, leg. SONG Jinxin (SWFU); **Myanmar:** 1 male, Carin Chebà, 900—1000m, L. Fea V XII—88/Museum Paris, ex. Coll. R. Oberthur (MNHN; dissected); 1 male, Carin Chebà, 900—1100m, L. Fea V XII—88 (MCSN; dissected); **Thailand:** 1 male, Mt. Doi Pui, 1400—1500m, Chiang Mai, N. Thailand, 13–V–1982, T. Shimomura leg. (MNHN; dissected); 1 female, same data but 16–V–1982 (MNHN); 1 male, Thailand, Chiangmai, Doi Pui, 26.V.1985 (RGCM; dissected); 1 famale, same data but 14.V.1985 (RGCM); 1 female, Thailand, Soppong Pai, 1—8.V.1993, Pacholatko & Dembicky leg. (RGCM); Laos: 1 female, LAOS-NE, Houa Phan prov., 20°12N, 103°59.5'E - 13.5'N, 104°01'E, Ban Saluei→Phou Pane Mt., 1340—1870m, 15.iv.-15.v.2008, Lao collectors leg. (NMPC).

##### Diagnosis.

Distinguishable from superficially similar specimens of *Tillicera auratofasciata* by body slender (length to width of elytra: male about 2.37, female 2.16), length to width ratio of terminal antennomere of both sexes equal to or above 1.5, metaepisternum and metasternum earth-yellow, pubescence on metaepisternum and metasternum white and distinctly thinner than light yellow decumbent setae on elytra, yellow setal pattern on elytral apical third much narrowing towards suture, dorsal and ventral sinuses of tegmen oblong, and dorsal margin of phallus with large denticles extending from apical third towards apex; and distinguishable from *Tillicera wenii* by pattern-forming setae on elytra more gold colored, meta-P1 absent and meta-P2 just vestigeal (Figs 39, 40), pit-like sensilla of male terminal antennomere larger and shallower [distinct in magnification 100x before treated with KOH solution] (Figs 30, 33), parameres parallel, with apex obtuse, dorsal and ventral sinuses oblong, and dorsal margin of phallus with large denticles extending from apical third towards apex.

##### Description.

Size: male: length 6.8–8.2 mm; width 2.2–2.4 mm; female: length 8.4–9.5 mm; width 2.8–3.0 mm.

Head: Black; clypeus, labrum, labium, maxillae yellow-brown, apex of palpi darker; antennomeres 1–3 paler; cranium with slightly dense, fine punctation, each puncture bearing a yellow or black seta; terminal antennomere: length to width ratio of both sexes > 1.5, male with a shallow pit-like sensillum at basal third, diameter 0.06 mm (distinct in magnification 100× before and after treated with KOH solution) (Figs 30, 32).

Prothorax: Pronotum bicolored, anterior to subapical depression black, posteriorly orange; with dense punctation and anteriorly directed black and yellow setae; prosternum orange.

Mesothorax: Mesoscutelllum orange; mesosternum earth-yellow.

Metathorax: Metasternum and metaepisternum earth-yellow, pubescence on metaepisternum and metasternum white, distinctly far thinner than light yellow decumbent setae on elytra.

Elytra: Length to width ratio: male average about 2.37, female average about 2.16; integument tricolored, basal third orange, posterior two-thirds black; with a pair of lateral yellow maculae before middle, each distance from lateral margin to two-thirds way towards suture, matted with golden yellow, decumbent setae; behind two maculae, with a central backward opened lunate band formed of yellow decumbent setae (sometimes with a pale integumental spot in middle); apical third with another pair of yellow maculae, spanning from lateral margin to, or nearly to, suture, matted with golden yellow, decumbent setae, setal pattern narrowing towards suture; basal third regularly and deeply punctate in rows, diameter of punctures larger than intervals; inner two rows of elytral punctation fading away behind lunate band; basal third with erect, black setae (extreme base with some yellow setae), black part with posteriorly directed, black, decumbent pubescence (apical third mixed with some yellow setae).

Legs: Black, paler at base, paler part of profemora not longer than one tenth, of mesofemora not longer than one fourth, of metafemora not longer than half; with moderately dense, grayish yellow, erect setae; all tibiae with distinct longitudinal carinae; pro- and meso- P1–4 present, P1 evident but unlobed, P2 feebly lobed apically, P3–4 conspicuously lobed apically; meta-P1 absent, meta-P2 just vestigeal and not clear, meta- P3–4 conspicuously lobed apically (Fig. 39).

Abdomen: Black.

Male terminalia: Ratio of length of paramere to whole tegmen 0.26: 1, parameres parallel, apex obtuse, dorsal and ventral sinuses both oblong, latter broader and deeper (Figs 13–14); phallic plate with large denticles along dorsal margin and fine granular structures on posterior sclerotized area (denticles extending from anterior third to posterior sclerotized area; number of denticles may vary between left and right phallic plate and between individuals but whole length of denticles is consistent) (Figs 15–16, 20); phallic ventral membrane with one line of file-like structures on each side (Fig. 15); spicular fork (Fig. 17); tergum VIII (Fig. 18), sternum VIII (Fig. 19).

Female reproductive organs: Vagina swollen, base of bursa copulatrix narrower than posterior end of vagina; length of bursa copulatrix three times as long as spermatheca; swollen zone between vagina and spermathecal duct smaller than that in *Tillicera sensibilis*; spermathecal gland attached to basal fourth of spermatheca (Fig. 38); both dorsal and ventral lamina of ovipositor divided.

##### Distribution.

China: Yunnan; Myanmar; Thailand; Laos.

##### Etymology.

The specific epithet *sensibilis* (= having the faculty of sensation) is a Latin adjective, referring to the presence of the pit-like sensillum in male terminal antennomere, which is first discovered in this species.

#### Tillicera wenii

G.Y. Yang & X.K. Yang sp. n.

urn:lsid:zoobank.org:act:305CC980-F9AB-4949-A794-9C7808F2E992

http://species-id.net/wiki/Tillicera_wenii

Figs 2 21–28 31 40 

##### Holotype.

male. Taiwan, Taoyuen country, Mt. Lalashan, 1994.V.10, leg. CHOU Wen-I. Specimen adhibitted on rectangular board; locality label (original in Chinese); label with information on dissecting number (108 #), dissector and date; holotype label; plastic vial with abdomen and aedeagus (NMNS). **Paratype**. 1 male, Taiwan, Hualien County, Bilyu Divine Tree, 1995.VI.19, leg. CHOU Wen-I (IZAS).

##### Diagnosis.

Distinguishable from superficially similar specimens of *Tillicera auratofasciata* by pattern-forming setae on elytra paler, metasternum  and metaepisternum earth-yellow, pubescence on metaepisternum and metasternum white and distinctly thinner than light yellow decumbent setae on elytra, meta-P1 evident and meta-P2 bilobed (Figs 39–40), pit-like sensilla of male terminal antennomere oblique, smaller and deeper (indistinct in magnification 100x before treated with KOH solution) (Figs 29, 31), paramere pointed; and distinguishable from *Tillicera sensibilis* by pattern-forming setae on elytra paler, meta-P1 evident and meta-P2 bilobed (Figs 39–40), pit-like sensilla of male terminal antennomere oblique, smaller and deeper (indistinct in magnification 100x before treated with KOH solution) (Figs 30–31, 33), paramere pointed, dorsal and ventral sinuses both lanceolate, and dorsal margin of phallus with large denticles extending from basal half towards apex.

##### Description.

Size: male: length 9.1–9.2 mm; width 2.5–2.6 mm; female: unknown.

Head: Black; clypeus, labrum, labium, maxillae yellow-brown, apex of palpi darker; antennomeres 1–4 paler; cranium with slightly dense, fine punctation, each puncture bearing a yellow or dark seta; terminal antennomere: length to width ratio > 1.5, males with an oblique and deep pit-like sensillum at basal third, diameter of opening 0.03 mm (indistinct in magnification 100x before treated with KOH solution) (Fig. 31).

Prothorax: Pronotum bicolored, anterior to subapical depression black, posteriorly orange, with some faint dark zones laterally; with dense punctation and anteriorly directed black and yellow setae; prosternum orange.

Mesothorax: Mesoscutelllum orange; mesosternum earth-yellow.

Metathorax: Metasternum and metaepisternum earth-yellow, pubescence on metaepisternum and metasternum white, distinctly far thinner than light yellow decumbent setae on elytra.

Elytra: Length to width ratio: male average about 2.30, female unknown; integument tricolored, basal two-fifths orange, posterior three-fifths black; with a pair of light yellow maculae before middle, each spanning from lateral margin to two-thirds way towards suture, matted with greyish yellow, decumbent setae; behind two maculae, with a central backward opened lunate band formed of yellow decumbent setae, more or less joining with anterior maculae; apical third with another pair of light yellow maculae, spanning from lateral margin to, or nearly to, suture, matted with greyish yellow, decumbent setae, setal patterns broadly meeting at suture; basal two-fifths regularly and comparatively shallowly punctate in rows, diameter of punctures a little smaller than, or as wide as, intervals; inner four rows of elytral punctation fading away behind lunate band; basal two-fifths with erect, black setae (extreme base with some light yellow setae), black part with posteriorly directed, black, decumbent pubescence (apical third mixed with some light yellow setae as well).

Legs: Black, paler at base, paler part of profemora not longer than one tenth, of mesofemora not longer than one fourth, of metafemora not longer than half; with moderately dense, grayish yellow, erect setae; all tibiae with distinct longitudinal carinae; P1–4 of all legs present, P1 evident but unlobed, P2 feebly lobed apically, P3–4 conspicuously lobed apically (Fig. 40).

Abdomen: Black.

Male terminalia: Ratio of length of paramere to whole tegmen 0.37: 1, paramere pointed, dorsal and ventral sinuses both lanceolate, former broader and deeper (Figs 21–22); phallic plate with large denticles along dorsal margin and fine granular structures on posterior sclerotized area; spicular fork (denticles extending from anterior half to posterior sclerotized area; number of denticles may vary between left and right phallic plate and between individuals but whole length of denticles is consistent) (Figs 23–24, 28); spicular fork (Fig. 25); tergum VIII (Fig. 26), sternum VIII (Fig. 27).

Female reproductive organs: Unknown.

##### Distribution.

China: Taiwan.

##### Etymology.

Dedicated to CHOU Wen-I, a specialist on Taiwan Cerambycidae and the collector of this species.

#### Tillicera bibalteata

Gorham, 1892 new record from China

http://species-id.net/wiki/Tillicera_bibalteata

Figs 41 45

Tillicera bibalteata  Gorham, 1892: 732 (Carin Hills, Chebà).

##### Diagnosis.

Differs from *Tillicera hirsuta* by absence of tubercular rows on basal elytral intervals, elytral apex not emarginate; from *Tillicera michaeli* by absence of tuberosity on elytral base (on third interval exactly) and absence of tubercular rows on basal elytral intervals, integument from which two bands of golden setae originate yellow, elytra apex not projecting nor acute, color of first three antennomeres light paler, legs black.

##### Distribution.

China: Yunnan, Sichuan, Hainan; Bhutan; Myanmar; Thailand; Laos; Vietnam; Cambodia.

##### Material examined.

 **Lectotype** designated by Gerstmeier & Bernhard (2010)**: ** female, “Tenasserim, Thagatà, Fea. Apr. 1887/Typus /*bibalteata*, Gorh./Syntypus, *Tillicera bibalteata*, Gorham, 1892 [handwritten by Raffaello Gestro]/Museo Civico di Genova/LECTOTYPE ♀, *Tillicera bibalteata*, Gorham, 1892, Revision 2009, Gerstmeier & Bernhard” (MCSN); **Paralectotype:** Carin Chebà, 900—1100 m, L. Fea V XII—88, Museo Civico di Genova (MCSN).

##### Other material examined.

12 specimens: 1 male, Yunnan, Jingdong, alt. 1170m, 1956.VI. 30, leg. Kryzhanovskij (IZAS); 1 female, Yunnan, Jingdong, Dongjiafen, alt. 1250m, 1956.VI.19, leg. A. Shnitnikov (IZAS); 1 male, Yunnan, Jinping, Mengla, alt. 500m, 1956.V.2, leg. HUANG Keren (IZAS); 1 male, Yunnan, Xiaomengyang, alt. 850m, 1957.V.3, leg. ZANG Lingchao (IZAS); 1 male, same data but leg. WANG Shuyong (IZAS); 1 male, Yunnan, Xishuangbanna, Mengpeng, alt. 550m, 1959.VI.27, leg. LI Suofu (IZAS); 1 female, Yunnan, Xishuangbanna, Damenglong, alt. 650m, 1958.V.4, leg. HONG Chunpei (IZAS); 1 female, Yunnan, Xishuangbanna, Menghun, alt. 1200–1400m, 1958.V.21, leg. MENG Xuwu (IZAS); 1 male, Yunnan, Mengla, Yaoqu, alt. 850m, 2005.V.11, leg. CUI Jianxin (CAU); 1 male, Sichuan, Chengdu, 1955.V.28, leg. HUANG Keren & JIN Gentao (IZAS); 1 female, Hainan, Shuiman, alt. 640m, 1960.V.25, LI Changqing (IZAS); 1 female, Mt. Doi Pui, 1400–1500m, Chiang Mai, N. Thailand, 20–V–1982, T. Shimomura leg. (MNHN).

#### Tillicera hirsuta

(Pic, 1926) new record from China

http://species-id.net/wiki/Tillicera_hirsuta

Figs 42 46

Thanasimus hirsuta  Pic, 1926: 22 (Tonkin).
Tillicera hirsuta : Gerstmeier & Bernhard, 2010: 18.

##### Diagnosis.

Differs from *Tillicera bibalteata* by having tubercular rows on basal elytral intervals, elytral apex emarginate; from *Tillicera michaeli* by absence of tuberosity on elytral base (on third interval exactly), diameter of interstitial tubercules larger than diameter of punctures on elytra, integument from which two bands of golden setae originates yellow, elytral apex emarginate, first four antennomeres paler.

##### Distribution.

China: Yunnan; Vietnam; Indonesia; Malaysia.

##### Material examined.

 **Lectotype** designated here: female, “Type [handwritten by Pic]/Lac Thô, Tonkin /*Thanasimus hirsutus*, n. sp. [handwritten by Pic]/Museum Paris, Coll. M. Pic/HOLOTYPE ♀ *Thanasimus hirsutus* Pic, 1926 = *Tillicera hirsuta*, Revision 2009, Gerstmeier & Bernhard” (MNHN).

##### Remarks. 

The name-bearing type was not fixed in the original publication and there was no imply how many type series was. For the purpose to fix the name-bearing type, the lectotype is designated here, which is the same specimen noted as "holotype" in Gerstmeier & Bernhard (2010).

##### Other material examined.

5 specimens: 1 female, Lac Thô, Tonkin (MNHN); 1 male, Phuyen binh, 1907 (MNHN); 1 female, Yunnan, Xishuangbanna, Menglun, Lvshilin, 21°54.61'N, 101°16.87'E, ca 630m, 2009.XI.14, leg. TANG Guo & YAO Zhiyuan, by fogging (IZAS); 1 male 1 female, Tonkin, Hoa-Binh, leg. A de Cooman (IZAS).

#### Tillicera michaeli

Gerstmeier & Bernhard, 2010 new record from China

http://species-id.net/wiki/Tillicera_michaeli

Figs 43 47

Tillicera michaeli  Gerstmeier & Bernhard, 2010: 23 (Laos).

##### Diagnosis.

Differs from *Tillicera bibalteata* by having a tuberosity on elytral base (on third interval exactly), each basal elytral interval with a row of tubercules, integument from which two bands of golden setae originate black, elytra apex slightly projecting and acute, color of first three antennomeres black, legs reddish brown; from *Tillicera hirsuta* by having a tuberosity in basediscal elytra (on third interval exactly), diameter of interstitial tubercules smaller than diameter of punctures on elytra, integument from which two bands of golden setae originate black, elytral apex slightly projecting and acute, first four antennomeres black.

##### Distribution.

China: Guangxi, Guangdong, Hainan; Vietnam; Laos.

##### Material examined.

 **Holotype:** male, “Laos, Phongsaly Prov., Phongsaly env., Phu Fa, h: 1450–1600 m/27.VII.2006, leg. M. Geiser, Bergregenwald, Umgestürzter Baum/HOLOTYPE male, *Tillicera michaeli* sp. n., Gerstmeier & Bernhard, 2009 Revision, Gerstmeier & Bernhard” (RGCM); **Paratype:** 2 specimens, same locality data as Holotype; 1 specimen, same data but 28.VII.2006 (RGCM).

##### Other material examined.

16 specimens: 1 male, Guangxi, Napo, Defu, alt. 1350m, 2000.VI.18, leg. LI Wenzhu (IZAS); 3 males, Guangdong, Nanling Nature Reserve, 2009.IV–VIII, leg. GAO Lei, by Malaise trap; 1 male, same data but by window trap; 1 male, same data but on soil; 3 males 2 females; same data but collecting methods unknown (IZAS). 1 male, Hainan, Yinggeling Nature Reserve, Hongxin village, 19.0805°N, 109.5210°E, alt. 415m, 2007.XII.3, leg. YANG Ganyan, by searching on dead rubber branches on wayside (IZAS); 1 male, same data but leg. WANG Zhiliang (IZAS); 1 female, Hainan, Jianfengling Nature Reserve, 1983.V.27, leg. Gu Maobin, by hand (IZAS); 1 male 1 female, Tonkin, Mt. Bavi, 800–1000m, 1941.VII, leg. A. De Cooman (IZAS).

## Discussion

The placement of the species *Tillicera wenii* sp. n. into the genus *Tillicera* might receive some controversy, because currently the most important character of the genus *Tillicera* is tarsal pulvillar formula 4–4–2 (Gerstmeier 2002, 2006; Gerstmeier and Bernhard 2010; Opitz 2010), while the tarsal pulvillar formula of *Tillicera wenii* is 4–4–4. But note that none of other species of genera related to *Clerus* and *Tillicera* (*sensu* Gerstmeier 2010) shows 4–4–4 tarsal pulvillar formula, and the general appearance, male phallus (phallic plate with large denticles along dorsal margin and fine granular structures on posterior sclerotized area) and presence of pit-like sensilla on male terminal antennomere suggest a close relationship among *Tillicera wenii* and *Tillicera auratofasciata* and *Tillicera sensibilis*, and its recent relatives might as well include *Tillicera soror* (from Bhutan) and *Tillicera javana* (from Java) based on their aedeagi illustrated by Gerstmeier and Bernhard (2010). “It is the genus that pronounces the characters, and not the characters that pronounces the genus” (Linnaeus 1737; Mayr 1969; Opitz 2010). *Tillicera wenii* might be a special and advanced species in the lineage of that includes *Tillicera auratofasciata*, *Tillicera sensibilis*, T. soror and *Tillicera javana*.

The extreme close relationship between *Tillicera auratofasciata* and *Tillicera sensibilis* can be additionally inferred from the characters below (unfortunately the female of *Tillicera wenii* is unknown): female vagina swollen, base of bursa copulatrix narrower than posterior end of vagina, length of bursa copulatrix three times as long as spermatheca, spermathecal gland attached to basal fourth of spermatheca; the male phallic posterior scleroitzed area of both sharply reduced in size, both have the ventral membrane with one line of file-like structures on each side, the pit-like sensillum on each terminal antennomere of males larger, shallower and straight.

*Tillicera bibalteata* and *Tillicera hirsuta* appear to be closely related; the evidence being that they have the same type of male phallus and female internal reproductive organs (phallic plate with small denticles on both dorsal and ventral margins; base of bursa copulatrix as wide as posterior end of vagina; length of bursa copulatrix is at least four times as long as spermatheca) (Figs 41–42, 45–46).

**Figures 3–4.**
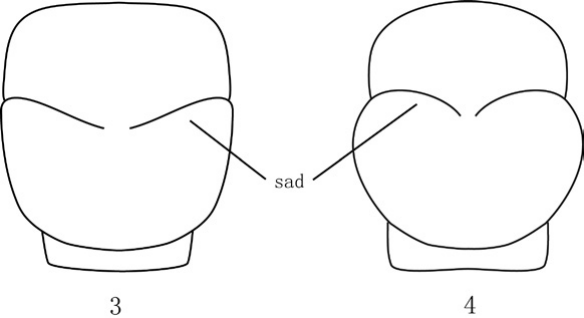
Pronotum **3** *Tillicera cleroides*, showing pronotum gradually narrowed after subapical depression **4** *Tillicera sensibilis* sp. n., showing pronotum swollen after subapical depression **sad** = subapical depression.

**Figures 5–12.**
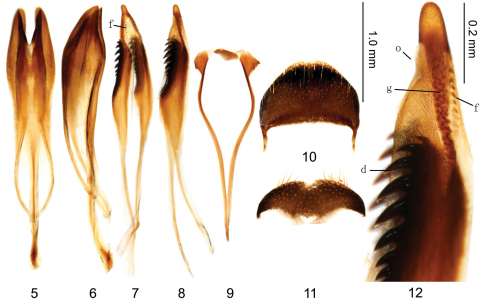
Male terminalia of *Tillicera auratofasciata*, from Tonkin **5–6** tegmen (**5** dorsal view **6** lateral view) **7–8** phallus (**7** ventral view **8** lateral view) **9** spicular fork **10** tergum VIII **11** sternum VIII **12** phallus, lateral view. Abbreviations: **d** = dorsal denticles **f** = file-like structures on phallic ventral membrane **g** = granular structures; **o** = median orifice.

**Figures 13–20.**
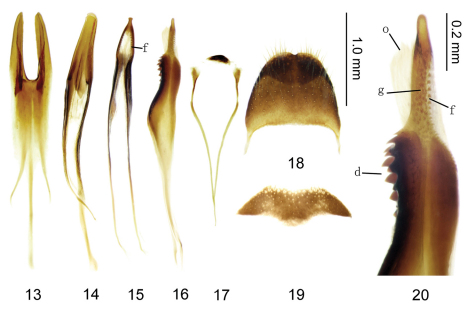
Male terminalia of *Tillicera sensibilis* sp. n., holotype **13–14** tegmen (**13** dorsal view **14** lateral view) **15–16** phallus (**15** ventral view **16** lateral view) **17** spicular fork **18** tergum VIII **19** sternum VIII **20** phallus, lateral view. Abbreviations: **d** = dorsal denticles **f** = file-like structures on phallic ventral membrane **g** = granular structures; **o** = median orifice.

**Figures 21–28.**
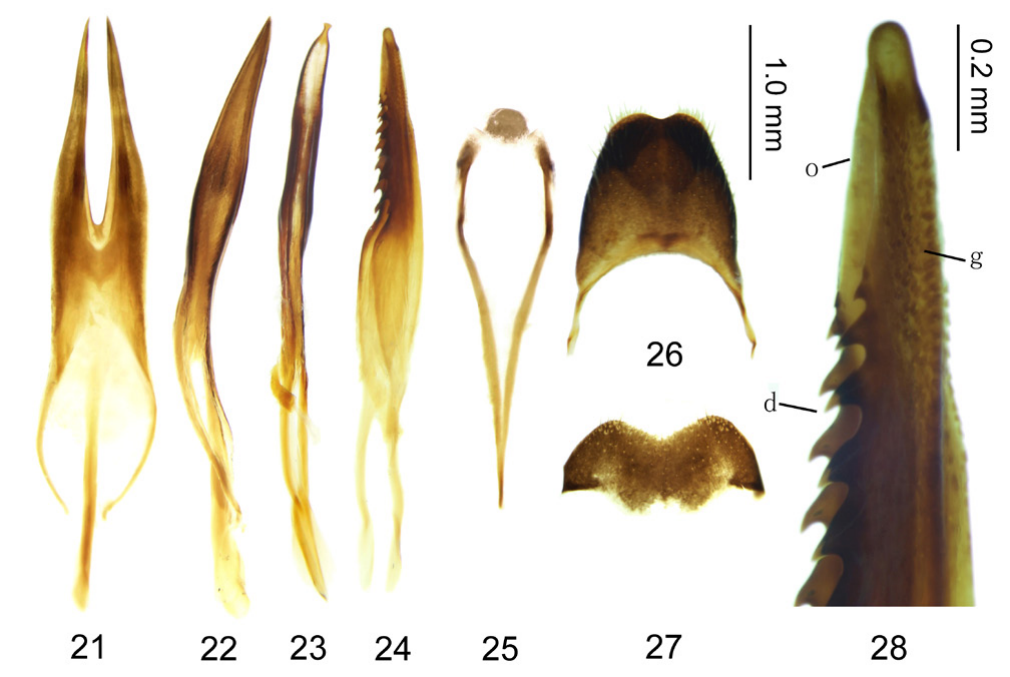
Male terminalia of *Tillicera wenii* sp. n., holotype **21–22** tegmen (**21** dorsal view **22** lateral view) **23–24** phallus (**23** ventral view **24** lateral view) **25** spicular fork **26** tergum VIII **27** sternum VIII **28** phallus, lateral view. Abbreviations: **d** = dorsal denticles **f** = file-like structures on phallic ventral membrane **g** = granular structures; **o** = median orifice.

**Figures 29–33.**
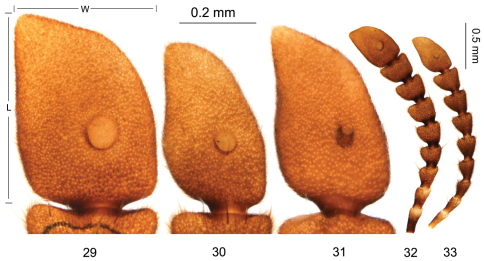
Male antennae in dorsal view, showing pit-like sensilla on terminal antennomere **29–31** terminal antennomere **29** *Tillicera auratofasciata*, from Guangxi **30** *Tillicera sensibilis*, sp. n., paratype **31** *Tillicera wenii*, sp. n., paratype **32–33** antennomere **3–11** **32** *Tillicera auratofasciata*, from Guangxi **33** *Tillicera sensibilis*, sp. n., paratype.

**Figures 34–38.**
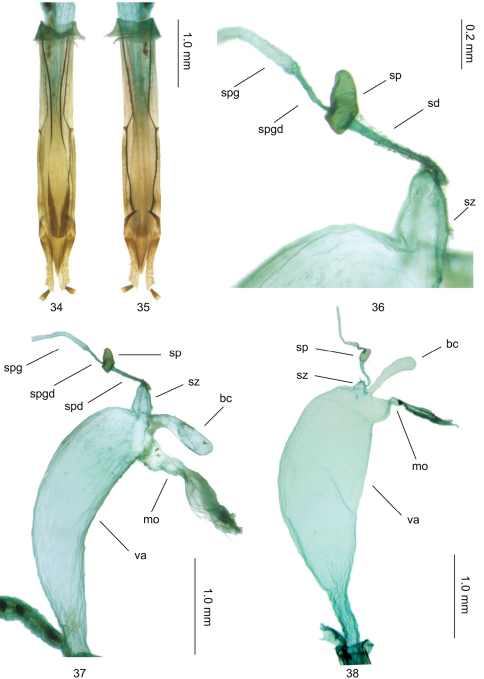
Female reproductive organs. **34–37** *Tillicera auratofasciata* from Yunnan **34** ovipositor, dorsal view **35** ovipositor, ventral view **36** spermatheca **37** internal organs in lateral view **38** *Tillicera sensibilis* from Yunnan. Abbreviations: **bc** = bursa copulatrix; **mo** = median oviduct; **sp** = spermatheca; **sz** = swollen zone; **spd** = spermathecal duct; **spg** = spermathecal gland; **spgd**= spermathecal gland duct; **va** = vagina.

**Figures 39–44.**
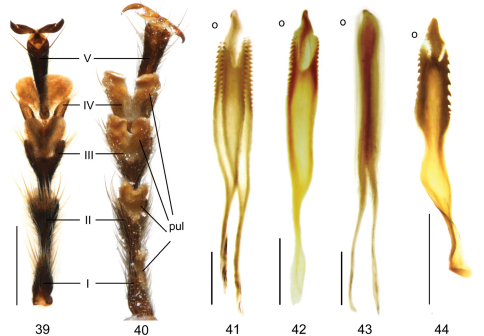
Metatarsus **39** *Tillicera sensibilis*, paratype, separated from body and treated with KOH **40** *Tillicera wenii*, holotype, left leg in natural condition. The Roman numerals indicate the segment of the tarsus. **pul** = pulvillus. Scale = 0.5mm **41–44** Phallus **41** *Tillicera bibalteata*, from Yunnan **42** *Tillicera hirsuta*, from Tonkin **43** *Tillicera michaeli*, from Hainan **44** *Tillicera cleroides*, from Hainan. **o** = median orifice. Scale = 0.5mm

**Figures 45–48.**
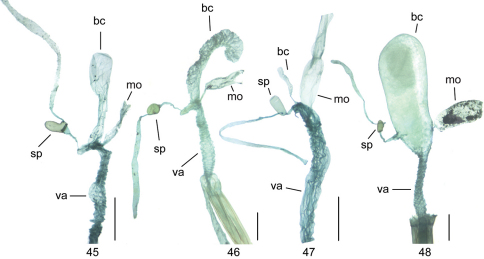
Female internal reproductive organs **45** *Tillicera bibalteata*, from Yunnan **46** *Tillicera hirsuta*, from Yunnan **47** *Tillicera michaeli*, from Guangdong **48** *Tillicera cleroides*, from Hainan. Abbreviations: **bc** = bursa copulatrix; **mo** = median oviduct; **sp** = spermatheca; **sz** = swollen zone; **spd** = spermathecal duct; **spg** = spermathecal gland; **spgd**= spermathecal gland duct; **va** = vagina.

The positions of *Tillicera cleroides* and *Tillicera michaeli* are uncertain. Phallus and female internal reproductive organs of *Tillicera cleroides* do not provide evidence of its relationship to other species (the phallic plate with small denticles along dorsal ventral margin and larger denticles on the ventral margin, base of bursa copulatrix as wide as posterior end of vagina, and spermathecal gland attached to the middle of spermatheca) (Figs 44, 48), but its pronotum indeed gives a very different look among this genus (Fig. 3). As for *Tillicera michaeli*, its phallic plate is uniformly sclerotized near the apex and without marginal denticles (Fig. 43), and the integument from which the two bands of golden setae originate is not yellow but black, which might indicate a close relationship to some species of *Clerus* than to other species of *Tillicera*.

Whether the current genus *Tillicera* is a monophyly might need further examination, because firstly the characters to support this genus currently (antennomeres triangularly dilated from the fifth and tarsal pulvillar formula 4–4–2) are not adequate; some species currently placed in *Tillicera* do not exhibit the typical triangularly dilated antennomeres and some Chinese related species not placed in *Tillicera* at present show 4–4–2 pulvillar formula as well. Secondly, as discussed in the above paragraph, some species currently placed in *Tillicera* might be closer to some species of *Clerus* than to other species of *Tillicera*. Actually, this is a problem of the generic definition of *Clerus*-*Tillicera* group, rather a problem of merely *Tillicera*. It is clear that a sound definition of the *Clerus*-*Tillicera* group, based on a greater range of characters (especially external and internal reproductive organs) is required, though this may not be accomplishable before all the representative species of this group are taxonomically revised. Conversely, the systematic importance of the pulvillar formula in *Clerus*-*Tillicera* group (especially for those with 4–3–2, 4–4–2 tarsal pulvillar formula) might require further test and the true systematic value of the pulvillar formula, however, may be better realized if these structures were more carefully considered in terms of the degree of development of each pulvillus rather than just a formula (eg. distinctly or feebly lobed apically, or present but unlobed or vestigeal).

The pit-like sensillum of the male terminal antennomere was discovered during the course of the present study, and we found this structure in some undetermined Chinese Cleridae as well. Whether this sexually correlated character can provide additional evidence to infer a close relationship between species-groups might require further investigation.

## Supplementary Material

XML Treatment for Tillicera auratofasciata

XML Treatment for Tillicera sensibilis

XML Treatment for Tillicera wenii

XML Treatment for Tillicera bibalteata

XML Treatment for Tillicera hirsuta

XML Treatment for Tillicera michaeli
